# Supplementary material for: MTNet: A combined diagnosis algorithm of vessel segmentation and diabetic retinopathy for retinal images
Source: PLoS One. 2022 Nov 23;17(11):e0278126. doi: 10.1371/journal.pone.0278126 (PMC9683560; doi:10.1371/journal.pone.0278126)
Supplement: Attachment — Submitted filename: Response to Reviewers.pdf [file pone.0278126.s001.pdf]

Song Gao
The School of Mechanical and Electrical Engineering,
Chengdu University of Technology
1#, Dongsanlu, Erxianqiao, Chengdu 610059, Sichuan, P.R.China
E-mail: gs@cdut.edu.cn
Aug 21, 2022

Dear Reviewer,

On behalf of my co-authors, I appreciate the reviewers very much for your positive and constructive comments and suggestions on our manuscript entitled “MTNet: A combined diagnosis algorithm of vessel segmentation and diabetic retinopathy for retinal images ” (ID: PONE-D-22-16068R1). We have studied the comments carefully and have made some revisions. We have tried our best to revise our manuscript according to the comments. Attached please find the revised version, which we would like to submit for your kind consideration.

Modifications to the original version are highlighted in 'Revised Manuscript with Track Changes'. The main corrections in the paper and the responses to the reviewer’s comments are as following:

Q1： It is mentioned in the page 12 that ASPP structure is used at the end of the CSP UNet Encoder side and behind each module in Decoder, and in figure 5 it is introduced that BottleneckCSP and ASPP are also used in UpsampleBlock which constitutes the decoder. Compared with figure 2, it is reasonable to think that the model uses BottleneckCSP in both the decoder and the encoder while ASPP is used twice at the beginning of the Decoder? Please unify the figure and text description.

A1： As the description in the manuscript caused confusion to the reviewer, we have revised the relevant illustrations and language. As shown below, the original diagram is on the left and the revised legend is on the right. In fact each UpsampleBlock consists of BottlenneckCSP, ASPP, and Upsample, as shown in Fig 5. The ASPP added to the 'Original image' is incorrectly represented, so we have modified the CSP_ UNet's overall structure to obtain the 'Modified image'.

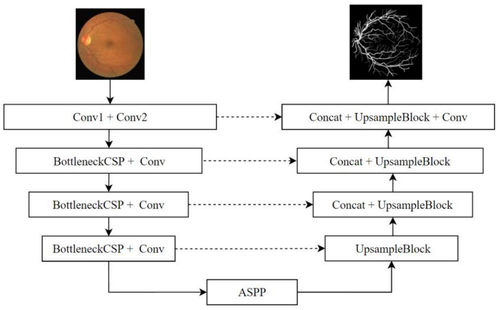

Original image

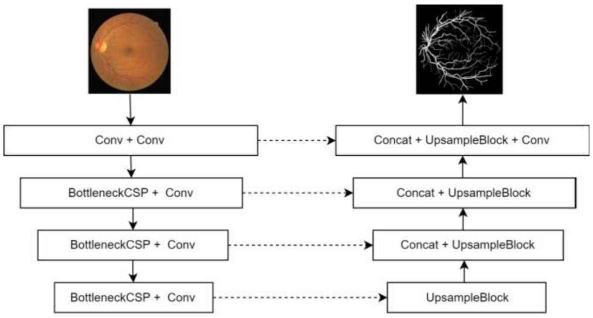

Modified image

Fig 2. Schematic diagram of CSP_UNet structure

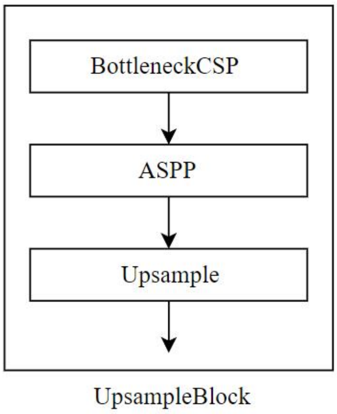

Fig 5. Structure diagram of UpsampleBlock module

Q2： It can be seen from Table 5 that AG-Net is better than CSP-UNet in terms of Precision, Recall, and has less number of parameters, could you please give the advantages of this model compared with the AG-Net?

A2： Based on the results in Table 5, we modified and added some descriptions on Page 11, Line 310-318 of the paper. Among several evaluation metrics, the more comprehensive evaluations of vessel segmentation performance are the IoU and the F1-score, and CSP-UNet has improved this two evaluation metrics compared to AG-Net and also has some advantages in terms of computational speed. Therefore, CSP-UNet is much better than AG-Net.

Table 5. Experimental results of CSP_UNet compared with other segmentation models

Model	IoU	Precision	Recall	F1-score	Accuracy	Parameters (MB)	GFLOPs
U-Net	0.6640	0.8003	0.9828	0.7981	0.9679	34.5	261.8
AG-Net	0.6645	**0.8060**	**0.9835**	0.7984	0.9682	**9.3**	65.7
DeepLab v3+	0.5664	0.7157	0.9748	0.7240	0.9555	59.3	88.7
R2 U-net	0.4941	0.6254	0.9634	0.6631	0.9429	39.1	611.2
UperNet	0.5751	0.7260	0.9759	0.7311	0.9569	126.0	182.1
CSP_UNet	**0.6674**	0.8030	0.9830	**0.8025**	**0.9683**	15.1	**46.0**

Q3： It's hard to tell the difference of this model from your explanation, please reorganize the language for Figure 8.

A3： We have modified Fig 8 and the associated language in the manuscript. Firstly, we have enlarged some details in Fig 8 to show more clearly the effect of CSP_UNet on retinal vessel segmentation. Secondly, we reorganised the description of Fig 8 in the manuscript (Page 12, Line 330-335). It was important to ensure that the revised language clearly reflected the differences between CSP_UNet and the others.

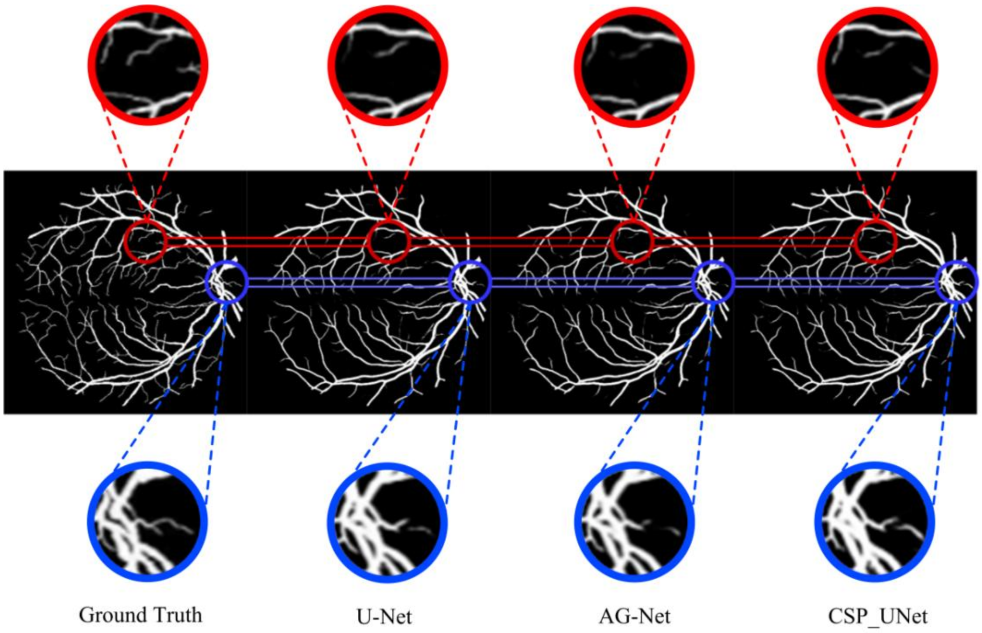

Fig 8. Comparison of segmentation details of CSP_UNet with U-Net and AG-Net

We tried our best to improve the manuscript and made some other changes in the manuscript. These changes will not influence the content and framework of the manuscript. We appreciate for Editor and Reviewers' warm work earnestly and hope that the correction will meet with approval.

Once again, thank you very much for your comments and suggestions.

Yours sincerely,

Song Gao
